# *Aloe vera*-Based Hydrogels for Wound Healing: Properties and Therapeutic Effects

**DOI:** 10.3390/gels9070539

**Published:** 2023-07-03

**Authors:** Mariana Chelu, Adina Magdalena Musuc, Monica Popa, Jose Calderon Moreno

**Affiliations:** “Ilie Murgulescu” Institute of Physical Chemistry, 202 Splaiul Independentei, 060021 Bucharest, Romania; mchelu@icf.ro (M.C.); pmonica@icf.ro (M.P.)

**Keywords:** *Aloe vera*, hydrogels, wound healing, wound dressings, therapeutic agents

## Abstract

*Aloe vera*-based hydrogels have emerged as promising platforms for the delivery of therapeutic agents in wound dressings due to their biocompatibility and unique wound-healing properties. The present study provides a comprehensive overview of recent advances in the application of *Aloe vera*-based hydrogels for wound healing. The synthesis methods, structural characteristics, and properties of *Aloe vera*-based hydrogels are discussed. Mechanisms of therapeutic agents released from *Aloe vera*-based hydrogels, including diffusion, swelling, and degradation, are also analyzed. In addition, the therapeutic effects of *Aloe vera*-based hydrogels on wound healing, as well as the reduction of inflammation, antimicrobial activity, and tissue regeneration, are highlighted. The incorporation of various therapeutic agents, such as antimicrobial and anti-inflammatory ones, into *Aloe vera*-based hydrogels is reviewed in detail. Furthermore, challenges and future prospects of *Aloe vera*-based hydrogels for wound dressing applications are considered. This review provides valuable information on the current status of *Aloe vera*-based hydrogels for the delivery of therapeutic agents in wound dressings and highlights their potential to improve wound healing outcomes.

## 1. Introduction

Medicinal plants have been used since ancient times. It has even been estimated that nearly 80% of the world’s population relies on traditional herbal medicine for primary health care [1]. Herbal therapies have recently shown an upward trend for a variety of ailments in parallel with the development of modern medicine. Many new drugs and treatments derived from medicinal plants are being developed and prescribed today. According to the World Health Organization (WHO), almost 25% of modern medicines are derived from plants that were used in traditional medicine. Additionally, many drugs are synthetic analogues obtained from model compounds isolated from plants [2]. This review summarizes the preparation, structural features, and properties of *Aloe vera*-based hydrogels and recent advances in *Aloe vera*-based hydrogels for wound dressing applications.

*Aloe vera* (*AV*) belongs to the Liliaceae family, of which the best-known species is *Aloe Barbadensis Miller*, and has been used for thousands of years in traditional medicine [3]. Being one of the most famous medicinal plants in the world, it is considered a miracle gift of nature due to its many therapeutic benefits [4].

References to the medicinal use of the *AV* plant date back 4000 years, but the first inscriptions mentioning the plant were found on a collection of Sumerian clay tablets from 2100 BC [5,6]. Additionally, in the Egyptian Ebers Papyrus of 1552 BC, the plant was mentioned as a laxative [5]. The first populations to identify and appreciate the healing properties of *Aloe* plants were the Egyptians, Romans, Greeks, Arabs, and Indians [7]. There were many legends, which said that the *Aloe* plant was used by the Egyptian Queen Nefertiti (1353 BC), considered “the most beautiful woman who ever lived”, and by Queen Cleopatra VII (69–30 BC) in their usual beauty treatments, but also as medicine. According to legend, in 333 BC, Aristotle advised Alexander the Great to capture the island of Socotra in the Indian Ocean for its famous *AV* plantations, which were needed to treat his wounded soldiers [5].

Starting in the 1950s, *Aloe* leaf gel began to be industrialized and commercialized. The global *AV* extracts market size is projected to grow from USD 2.65 billion in 2023 to USD 4.55 billion by 2030 at a compound annual growth rate of 8.0% during the forecast period [8]. The market demand for *AV* products is now widespread globally and has been steadily increasing, driven by consumer awareness of its various health benefits associated with medicinal and cosmetic properties and the growing preference for natural and organic herbal products, including (i) health and wellness products such as dietary supplements, herbal remedies, and functional beverages, for their potential health benefits such as aiding digestion and supporting the immune system; (ii) skin care products and cosmetics, e.g., lotions, creams, gels, and face masks, due to its soothing and moisturizing properties; (iii) pharmaceuticals: *AV* extracts are used in the production of ointments, creams, and oral medications for burns, wounds, psoriasis, and gastrointestinal disorders; (iv) agriculture and farming: *AV* is used in soil improvement and as a natural fertilizer. Gel-based pharmaceutical and skin care products account for approximately 80% of the market size.

*AV* is a shrubby plant with fleshy green leaves, conical and filled with a clear, viscous gel. It grows perennially in many areas of the globe [9,10]. *AV* gel has been used for curative and therapeutic purposes, and numerous bioactive components have been discovered in the inner gel. It was believed that the special biological activities of *AV* gel are due to the synergistic effect of the multitude of biochemical components present in its composition. It exhibits numerous biological benefits such as astringent, anti-diabetic, anti-ulcer, antibacterial, anti-inflammatory, antimicrobial, antioxidant, hemostatic, and anti-carcinogenic properties and also effectiveness in treating gastrointestinal disorders [11,12].

*AV* is a plant often cultivated in people’s homes around the world as a natural compound intended for widespread use by both adults and children and recognized in clinical practice as a tool for wound healing [13,14,15,16,17]. *AV* gel has been particularly associated with the treatment of skin injuries such as cuts, burns, frostbite, radiation, and electrical injuries [18,19,20,21].

Depending on the evolution of the recovery process, wounds can be classified into two broad categories: acute and chronic wounds [22,23]. Acute wounds are injuries with complete healing within up to 12 weeks [24,25]. In contrast, chronic wounds take more than three months to heal. This may be due to repeated tissue damage or associated physiological conditions such as poor primary treatment, infections, diabetes, malignancy, severe injury, or a compromised immune system [26,27,28].

Wound care is necessary to prevent or mitigate possible infection, the most common complication for compromised skin. Dressings are mainly applied to prevent microorganisms from reaching the wound, to keep the wounded area hydrated, and to absorb exudates [29,30]. Traditionally, sterile gauze dressings have been widely applied to wounded areas [31,32,33]. However, they are not always effective because they do not provide hydration, and sometimes their removal becomes painful because they stick to the wounds. Additionally, to prevent the development of infections, different creams and ointments with antimicrobial action are used, which must be removed and reapplied constantly [34,35,36]. Modern dressings are adapted to different types of injuries and patient typologies to avoid infection and promote scarless healing. They are designed to provide hydration and interact with wounds by releasing bioactive molecules to accelerate the wound-healing process [37,38].

With the adaptation of synthesis methods and the evolution towards ecological chemistry, it is absolutely necessary to use non-toxic solvents for the production of dressings. Thus, dressings such as dermal patches, foams, hydrogels, hydrocolloids, nanoparticles, nanofibers, films, membranes, and three-dimensional (3D) printed scaffolds can be obtained with various bio-based adaptive features [39,40,41,42,43,44].

Hydrogels are a class of materials often applied in the soft tissue engineering of skin, blood vessels, and muscles [45,46]. With a three-dimensional porous structure, hydrogels are formed by physically or chemically crosslinked bonds of hydrophilic polymers [47,48,49]. They are also insoluble and have an exceptional capacity to absorb wound exudates and allow oxygen diffusion to accelerate healing [25,50,51,52]. They can retain several times more water compared to their dry weight and maintain good hydration in the injured area [53,54]. Due to these unique physical properties, hydrogels are the most suitable dressings to cover skin wounds [55,56,57]. Hydrogel design and development can provide a platform for the encapsulation of cells, antibacterial agents, or bioactive factors. As dressings, hydrogels must be biocompatible, have suitable physical and mechanical properties, and ensure cell proliferation in wounds [58,59,60].

Throughout history, humans have used native *AV* gel, which has been shown to have exceptional properties in the wound-healing process and in promoting tissue regeneration. The huge potential of *AV* gel is due to the advantages of the biocompatible, bioavailable, and biodegradable matrix, as well as the ability to heal wounds easily and effectively without leaving scars [52,61,62]. Native *AV* gel not only releases bioactive components but also moisturizes the wound to increase flexibility, acts as a barrier against foreign microbes, and helps reduce pain at nerve endings [21].

## 2. Phytochemical Constituents of *Aloe vera*

**Figure 1:**
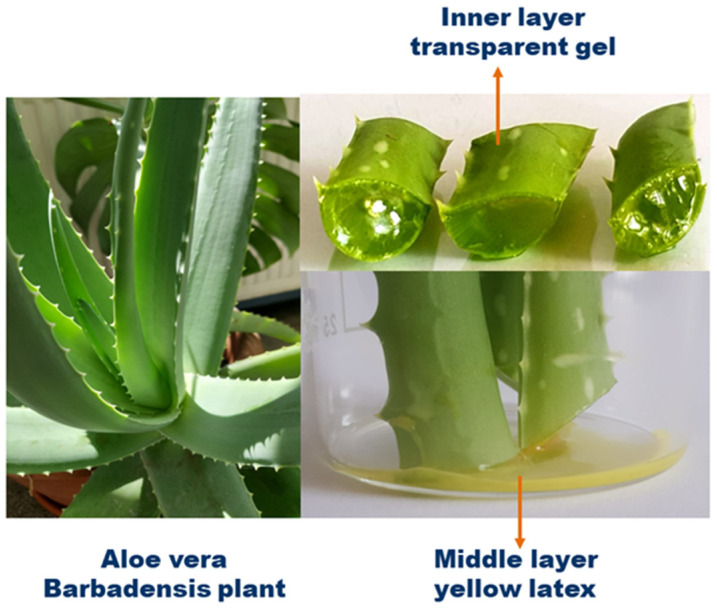
Raw morphology of the *Aloe vera* plant.

Numerous studies have demonstrated the exceptional healing potential of *AV* and identified the many bioactive compounds responsible for wound healing. The structure of the *Aloe* leaf is configured in the form of three layers. The inner layer consists of a transparent gel containing 99% water and 1% solid matter that compresses over 75 different compounds (such as glucomannans, amino acids, lipids, sterols, and vitamins), the middle layer is a bitter latex in the form of yellow juice rich in glycosides and anthraquinones, and the outer layer is a thick cortex that produces carbohydrates and proteins (Figure 1) [63,64,65,66,67].

Depending on the species, the influence of climatic conditions, and the diversity of the ecosystem to which they belong, the phytochemical constituents can be different in *AV* plants. Harvested from the inside of the leaves of the *AV* plant, the gel is a gelatinous substance that contains a complex variety of several bioactive compounds, and the analysis of the dry matter of the dry *AV* gel showed that it mainly contains polysaccharides (approx. 55%), sugars (approx. 17%), minerals (approx. 16%), proteins (approx. 7%), lipids (4%), and phenolic compounds (approx. 1%) [4,68,69,70,71,72]. One of the most important compounds of the gel is acemannan, which is used in many pharmacological and biological applications in medical and industrial fields, such as dentistry [73], metabolic disorders [74], cardiovascular diseases [75], and tumor diseases [76]. It has also been used for wound treatment [77] and drug delivery [78,79]. Other constituents, such as amino acids, are building blocks for body and muscle proteins; sugars control cholesterol levels, proper digestion, liver function, and help strengthen bones. Anthraquinones have an antiviral effect, enzymes catalyze the biochemical reactions, inorganic compounds have a role in the proper functioning of several enzymes in various metabolic pathways, vitamins have a strong antioxidant action in neutralizing free radicals, proteins have an antitumor effect, and hormones and sterols promote wound healing.

**Figure 2:**
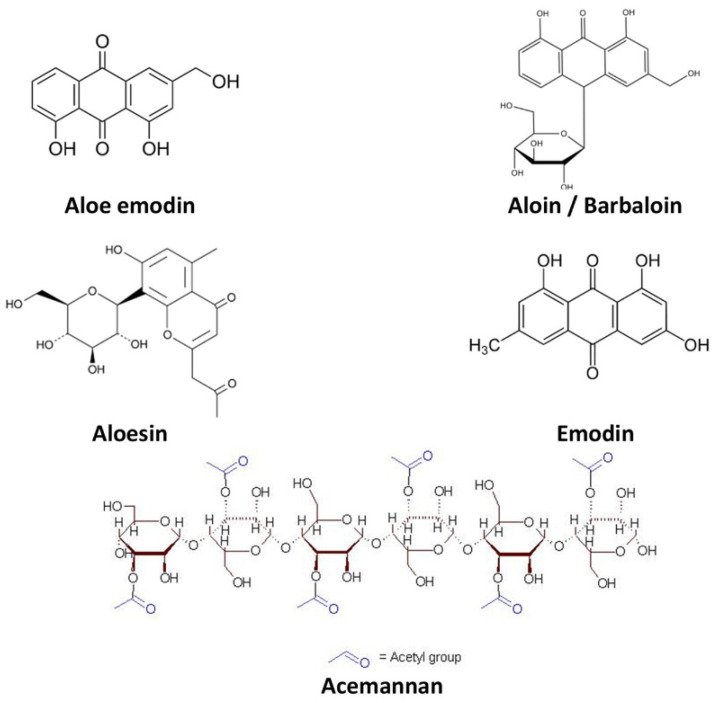
Chemical structure of the main biocomponents isolated from *Aloe vera* [68].

It is believed that the power to adjust the various biological and therapeutic implications of *AV* gel is due to the synergistic effect of all the active phytochemical components. This unique composition enabled the gel to harmoniously integrate into human tissues, promoting natural healing and regeneration processes. Applied topically to a wound, *AV* gel acts gently but as a potent antimicrobial and anti-inflammatory agent, inhibiting bacterial growth and reducing inflammation [21,80,81,82,83,84]. Table 1 summarizes the main biocomponents of *AV*. Additionally, the active compounds of the gel stimulate the production of new cells and collagen, which is an essential protein in the process of tissue regeneration (Figure 2) [68,85]. Thus, wounds treated with *AV* gel heal faster and without leaving unsightly scars.

## 3. Preparation of *Aloe vera* Hydrogels

**Figure 3:**
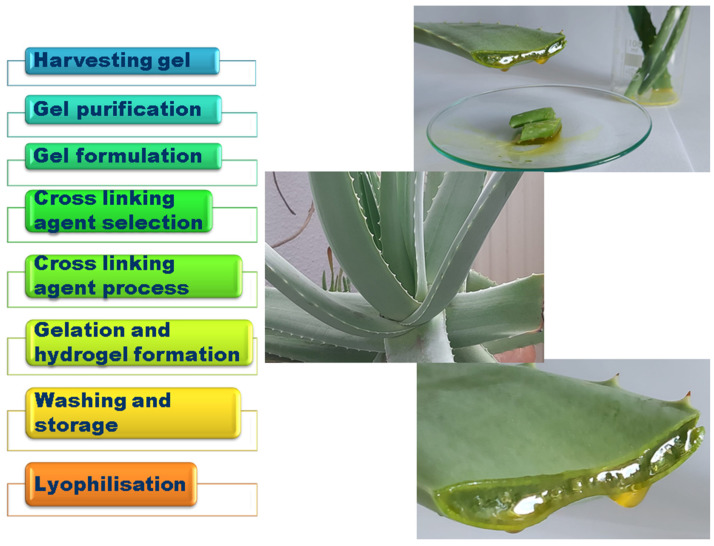
Scheme of the preparation of *Aloe vera* gel.

**Figure 4:**
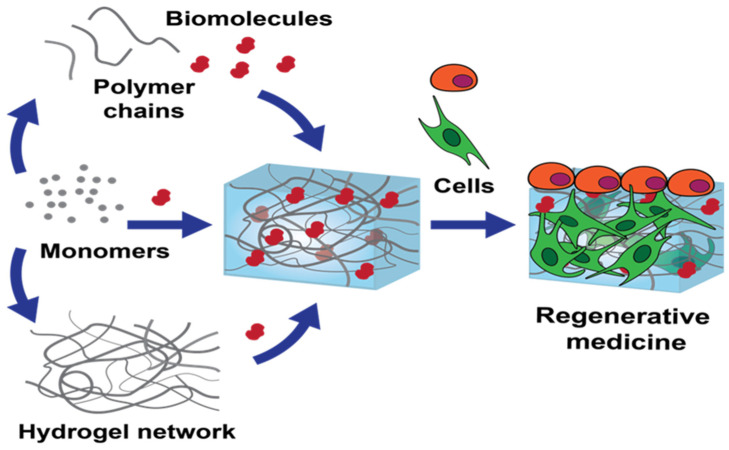
Graphical representation of *Aloe vera* hydrogel network preparation [86].

*AV* gel can serve as a natural and biocompatible matrix for hydrogel. It can be obtained by extracting the gel from mature *AV* leaves that are healthy and free from any damage or discoloration, removal of the yellow latex layer, which can be irritant, and processing the clear gel in the inner leaf to remove any impurities by washing with distilled water or ethanol. After purification and excess water draining (a concentration of 1–10% (*w*/*v*) is typically used for hydrogel formulations), the gel can be mixed with a cross-linking agent, such as a suitable polymer, considering factors such as gelation time, biocompatibility, and stability of the cross-linked hydrogel, to form a hydrogel. Finally, the gel is washed with distilled water to remove any unreacted cross-linking agent or by-products and stored refrigerated in a moisture-sealed container to maintain its moisture content (Figure 3). In Figure 4, the procedure for the *AV* hydrogel network preparation for its use in regenerative medicine is represented [86,87].

The specific procedure for preparing *AV*-based hydrogels can vary depending on the desired application and the chosen cross-linking method. It is essential to follow good laboratory practices and refer to relevant literature or established protocols to ensure the reproducibility and quality of the hydrogel preparation. It is worth mentioning that the incorporation of therapeutic agents, such as antimicrobial and anti-inflammatory agents, into *AV*-based hydrogels can enhance their potential for wound healing and other biomedical applications. By combining *AV* medicinal properties and wound healing effects with the controlled release capabilities of hydrogels, it is possible to develop advanced biomaterials with improved therapeutic outcomes. Therapeutic agents can be added to the *AV* gel solution before or during the cross-linking process. This can be achieved by dissolving the agents in a suitable solvent and then mixing them with the gel solution. The concentration of the agents can be varied to control the release rate and dosage. Additionally, the incorporation of therapeutic agents can be attained by the selection of appropriate therapeutic agents with desired antimicrobial and anti-inflammatory effects based on the specific application. Examples of antimicrobial agents include silver nanoparticles [88], antibiotics [89], or natural antimicrobial compounds [90], while anti-inflammatory agents may include corticosteroids [91] or non-steroidal anti-inflammatory drugs (NSAIDs) [92].

**Figure 5:**
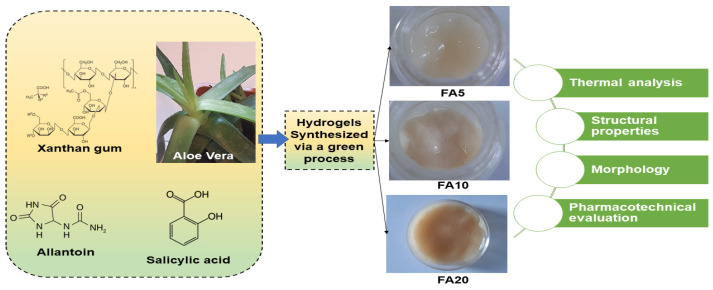
Synthesis, optical imaging of three composite hydrogels with different concentrations of *Aloe vera* (5%, 10%, and 20% *w*/*v*), and evaluation of their properties [94].

The cross-linking and gelation of *AV* hydrogels are crucial steps in the preparation process to convert the AV gel solution into a solid hydrogel matrix. Cross-linking is the process of creating covalent or physical bonds between polymer chains, resulting in a three-dimensional network that gives the hydrogel its structural stability and enhanced mechanical properties. Gelation refers to the transformation of the liquid gel solution into a solid gel form. The gelation process involves mixing the *AV* gel solution with an appropriate concentration of cross-linking agent and allowing it to react for a specific period. The *AV* gel solution containing therapeutic agents can be cross-linked using a suitable method, such as chemical cross-linking or physical cross-linking. Cross-linking agents are substances that promote the formation of covalent bonds between polymer chains, resulting in a three-dimensional network structure. This network improves the gel’s strength, elasticity, and resistance to dissolution in aqueous environments, making it suitable for wound healing applications. Various cross-linking mechanisms and agents can be utilized for *AV*-based hydrogels. Chemical cross-linking may involve the addition of a cross-linking agent that reacts with hydroxyl groups in *AV* to form covalent bonds, leading to gelation; while physical cross-linking can be achieved through temperature, pH-incorporating temperature, or pH-responsive polymers, the hydrogel forms as the polymer chains undergo a conformational change by simply heating the gel solution to a specific temperature or adjusting the pH. Certain polymers, such as alginate, can undergo ion-induced gelation in the presence of divalent cations such as calcium ions. Calcium chloride (CaCl_2_) is commonly used to initiate gelation in *Aloe vera*-alginate composite hydrogels. The gelation occurs as the calcium ions form ionic cross-links with the alginate chains [93]. A few commonly employed cross-linking methods are: (i) temperature-induced gelation: *AV* polymers can undergo gelation when the temperature is raised above a critical point, forming a physical cross-linked network; (ii) ionic gelation: addition of multivalent cations, such as calcium ions (Ca^2+^), can induce gelation by creating ionic interactions between the *AV* polysaccharides; (iii) natural agents such as glutaraldehyde, genipin, and tannic acid can be used to chemically cross-link *AV* hydrogels, these agents react with the functional groups present in the polymer chains, forming stable covalent bonds; (iv) carbodiimides such as 1-ethyl-3-(3-dimethylaminopropyl) carbodiimide (EDC), can facilitate the formation of amide bonds between carboxylic acid groups of *AV* polymers and amine groups from other molecules, resulting in cross-linking; (v) radiation-induced cross-linking: hydrogels can be cross-linked using ionizing radiation, such as gamma rays or electron beams, these high-energy radiations cause the formation of free radicals within the polymer chains, leading to cross-linking. These cross-linking mechanisms and agents help improve the mechanical integrity, swelling behavior, and biocompatibility of *AV*-based hydrogels used in wound dressings. They promote the stability of the hydrogel structure, prevent rapid dissolution in contact with wound exudate, and ensure the sustained release of beneficial components from *AV* for wound healing purposes. It is important to note that the specific choice of cross-linking agent and method may depend on factors such as desired properties, safety considerations, and compatibility with the wound healing environment. During cross-linking and gelation, it is important to control parameters such as temperature, pH, and reaction time to achieve the desired gel properties. The gelation time can be influenced by factors such as the concentration of cross-linking agents, AV gel concentration, and the specific method used. It is crucial to optimize these parameters to obtain hydrogels with desirable properties, such as mechanical strength, swelling behavior, and drug release characteristics. After gelation, it is common to wash the hydrogel to remove any unreacted cross-linking agents or by-products. The resulting *AV* hydrogel can be characterized and evaluated for its physical, chemical, and biological properties, such as gelation time, swelling behavior, mechanical strength, and drug release profile. In vitro and in vivo studies can be conducted to assess the antimicrobial and anti-inflammatory efficacy of the hydrogel, as well as its biocompatibility, to ensure its suitability for various applications, including wound healing, drug delivery, and tissue engineering. Figure 5 presents a schematic illustration of the synthesis and characterization of three composite hydrogels with different concentrations of *AV*, 5%, 10%, and 20% (*w*/*v)*, and the assessment of their properties [94]. The natural polymer-based hydrogels with high *AV* content, from 38% to 71% by weight in dry gel, demonstrated improved pharmacotechnical properties, including swelling ratio, spreadability, elasticity, and tensile strength. The hydrogel with *AV* content of 10% (*w*/*v*) in solution and 55% by weight in dry gel exhibited the highest strength, elasticity, and absorption capacity and also a slightly higher spreadability, indicating it for application in wound care [94].

## 4. Biological and Pharmacological Effects of *Aloe vera*

AV gel has multiple functions. It can be used in the food field due to its proven biological properties such as antioxidant, antiviral, antibacterial, antifungal, and antiochratoxigenic activity against *Aspergillus carbonarius*, *Aspergillus niger*, *Penicillium digitatum*, *Penicillium expansum*, and *Botrytis cinerea* [63,95,96,97,98]. It is widely used to produce gel-containing healthy drinks and juices, including sports drinks [99]. It can be a functional food in the activation of lipolysis and the prevention of metabolic changes related to obesity since the phytosterols of *Aloe* gel are effective in reducing visceral fat due to the interaction with cholesterol and also has an effect on glucose metabolism, reducing blood sugar in the experimental mouse model [100]. It acts in intestinal disorders (combats constipation) due to its laxative, anti-dysenteric, anti-hemorrhoidal, and cicatrizing properties [101,102,103]. Moreover, even *AV* flowers are consumed more often today, knowing that diets rich in antioxidants reduce the risks of cardiovascular diseases and cancers [104]. 

Additionally, *AV* gel can be used in the medical field due to its demonstrated pharmacological effects on several components of the metabolic syndrome, such as effects against dyslipidemia, hyperglycemia, hypertension, and obesity [105]. Numerous studies have highlighted the beneficial anti-inflammatory, anti-diabetic, immunomodulatory, and anticancer (neoplastic disease) capacity [106,107,108].

At the same time, it has been studied for its active capabilities, such as hepato-protective, anti-ulcer, anti-arthritic, and anti-rheumatic properties [109,110,111]. Many investigations have shown that the dental uses of *AV* are multiple, with a positive impact on the oral area [112,113,114]. In the case of broken, avulsed teeth, the extract (50%) of *AV* determined the increase in the cell viability of the stem cells in the dental pulp. This result is due to polysaccharides and especially acemannan, which have a positive effect on the growth factor, the expressions of specific osteogenic genes, and DNA synthesis [115,116]. 

**Figure 6:**
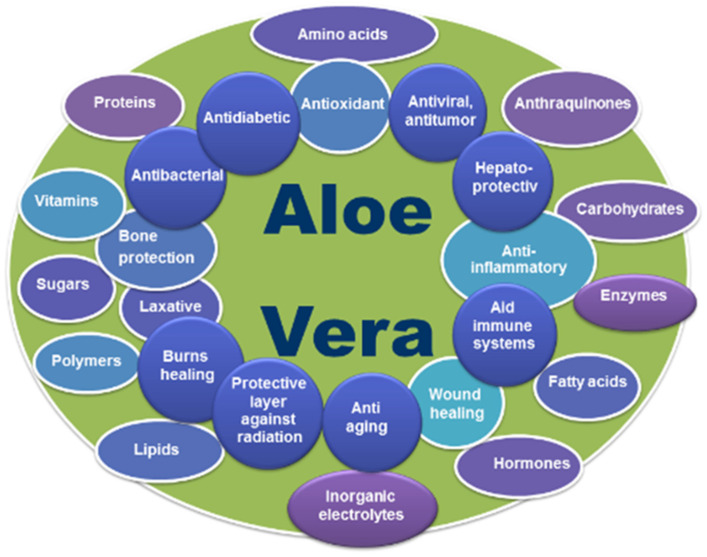
Graphical representation of the correlation between properties and composition of *Aloe vera*.

*AV* has a crucial contribution in reducing pain, combating inflammation, moisturizing the wound, improving the quantitative and qualitative composition of collagen, and improving the migration of neighboring epithelial cells of the wound [117]. *AV* has valuable pharmaceutical properties both through the contained gel and the whole leaf extract, which include the possibility of co-administration of bioavailable vitamins to humans. In a study on human subjects, *Aloe* was found to increase the absorption of both vitamins C and E through a slower absorption mechanism, and the vitamins last longer in plasma with *Aloe. Aloe* is said to be the only supplement known to improve the absorption of both vitamins and should be considered a true supplement [118]. Figure 6 presents a graphical representation of the interrelationship between the properties and composition of *AV*. 

The versatile nature of *AV* gel has significant potential in the field of pharmaceutical applications, particularly in improving the absorption capabilities of poorly absorbed orally administered drugs. Different formulations can encapsulate poorly absorbed drugs, while *AV* gel acts as a stabilizing and enhancing agent [119,120,121]. Due to its outstanding efficacy and compatibility with different drug carriers, the use of *AV* can be further expanded in potential applications and provides a flexible platform for optimizing oral drug delivery.

The release of therapeutic agents from *AV*-based hydrogels can occur through several mechanisms, including diffusion, swelling, and degradation of the hydrogel matrix. These mechanisms play a crucial role in controlling the release rate and duration of the therapeutic agents. Here is an overview of these mechanisms:

*Diffusion-controlled release*: Diffusion is the most common mechanism for the release of therapeutic agents from hydrogels. The hydrogel matrix acts as a barrier, and therapeutic agents diffuse through the gel network. The release rate is governed by the concentration gradient between the hydrogel and the surrounding medium. The diffusion coefficient of the therapeutic agent in the hydrogel matrix, as well as the pore size and structure of the hydrogel, influence the release kinetics. Factors such as the molecular weight and solubility of the therapeutic agent also affect diffusion-controlled release [122]. 

*Swelling-controlled release*: *AV*-based hydrogels have the ability to absorb water and swell, affecting the release of therapeutic agents. When the hydrogel comes into contact with an aqueous medium, it absorbs water and swells, leading to an expansion of the gel network. The swelling of the hydrogel creates channels or pores, facilitating the release of therapeutic agents. The release rate depends on the degree of swelling, which can be influenced by factors such as hydrogel composition, cross-linking density, and environmental conditions (e.g., pH and temperature) [123].

*Degradation-controlled release*: Some *AV*-based hydrogels can undergo controlled degradation over time. The hydrogel matrix degrades through processes such as hydrolysis, enzymatic degradation, or biodegradation, leading to the release of therapeutic agents. The degradation rate is influenced by factors such as the composition of the hydrogel, cross-linking density, the presence of enzymes or catalysts, and the physicochemical environment. As the hydrogel degrades, the therapeutic agents are gradually released into the surrounding medium [124,125].

These release mechanisms can occur individually or in combination, depending on the specific formulation and properties of the *AV*-based hydrogel, as well as the characteristics of the therapeutic agents. The choice of cross-linking agents, gel composition, and hydrogel architecture can be tailored to optimize the release profile, achieving sustained or controlled release over a desired period. The release of kinetics can also be influenced by external factors such as temperature, pH, and mechanical forces. Additionally, the interactions between the therapeutic agents and the hydrogel matrix, such as electrostatic or chemical interactions, can also impact the release behavior. Therefore, it is essential to carefully design and characterize *AV*-based hydrogels to achieve the desired release profile for specific therapeutic applications.

*AV*-based formulations have both inhibitory and stimulatory properties that can influence inflammatory processes and wound healing. Its inhibitory system refers to its capacity to reduce inflammation and exhibit anti-inflammatory activity. On the other hand, its stimulatory system refers to its power to promote wound healing. Together, these dual systems allow *AV* to modulate the complex interplay between wound healing and inflammation beneficially. Both the native gel and hydrogels based on *AV* showed beneficial effects and proved effective in different applications, in oral and topical therapies. They accelerate the rate of wound closure and skin healing and alleviate mucocutaneous problems, including gingivitis. As a natural medicine, it is used in oral mouthwashes, toothpaste, submucosal fibrosis, vaginal atrophy in menopausal women, and mucosal lesions induced by chemotherapy and radiotherapy or in veterinary practice. Here, we highlight some main beneficial effects *of AV* hydrogels in wound healing.

### 4.1. Reduction of Inflammation 

Psoriasis is an immune disease, provoked by an unclear cause, which is characterized by inflammation caused by the dysfunction of the immune system and is manifested by an itchy rash, most commonly on the knees, elbows, trunk, and scalp. This disease can cause inflammation in the body and can also affect other organs or tissues in the body. Worldwide, approximately 125 million people suffer from this disease. Plaque psoriasis is associated with several comorbidities, including inflammatory arthritis, cardiometabolic disease, and depression. The American Academy of Dermatology—National Psoriasis Foundation guidelines recommend biologics as alternatives for the first-line treatment of moderate to severe plaque psoriasis due to their therapeutic efficacy and acceptable safety profiles [126]. *AV* has often been used for topical applications in the treatment of psoriasis. A study on rats, in which hydrogels based on *AV* mucilage were developed and prepared with 80% *w*/*w* of gel for topical applications, demonstrated good efficiency in controlling hyperkeratinization, showing a 61% reduction of the stratum corneum on the tested animals. The results confirmed the keratolytic action of *AV* hydrogel, which can be used to treat psoriasis. The effect of *AV* leaf extract has been attributed to polysaccharides, rich in glucomannan and acemannan, pectic compounds, cellulose, and hemicelluloses, which determine most of the plant’s therapeutic properties [127]. The antipsoriatic properties of *AV* have been combined with the healing activity of Natural Rubber Latex to produce new economic occlusive dressings recommended for the treatment of psoriasis symptoms. In total, 58.8% of loaded *AV*, present on the surface and inside the dressing, was released after 4 days. An in vitro study on human dermal fibroblasts and sheep blood, respectively, confirmed the biocompatibility and hemocompatibility of the new dressings, the preservation of approximately 70% of the free antioxidant properties of *AV*, and the total content of phenolic compounds 2.31 times higher in these dressings compared to natural rubber latex without *AV* [128].

### 4.2. Prevention of Bacterial Infection

Chitosan and *AV* films encapsulating thymol were prepared to be used in preventing the possibility of bacterial infection and showed a high thymol encapsulation efficiency of 95.3% with good dispersibility. Test results against various pathogenic microbes such as *Bacillus*, *Staphylococcus*, *Escherichia*, *Pseudomonas*, *Klebsiella*, and *Candida* showed that the films were effective against bacterial colonization in a thymol concentration-dependent manner. The addition of *AV* increased the water absorption of the films, which is one of the primary factors of healthy wound healing and helped by improving the antioxidant activity and in vitro release efficiency of thymol [129]. New polymer composite films based on polyvinyl alcohol and *AV* have been prepared for wound healing and prevention of surgical wound infections. Films tested for antibacterial and antifungal activity against *E. coli*, *P. aeruginosa*, *Aspergillus flavus*, and *Aspergillus tubingensis* showed antimicrobial activity against all strains; the lowest concentration of *AV* (5%) showed the highest activity against all strains. Sutures of wounds covered with films based on polyvinyl alcohol and *AV* showed that the new composites have antibacterial effects and the potential to be used in the prevention of infections at the surgical site and can be used for wound healing purposes [130]. Films based on alginate, *AV* gel, honey, and cellulose nanocrystals can be used for applications as antibacterial dressings. The morphological, swelling, mechanical, and biological properties of the films prepared and tested against the Gram-negative organisms *Salmonella typhi*, *Klebsiella pneumoniae*, *Escherichia coli*, and the Gram-positive organism *Staphylococcus aureus* were estimated. The films showed superior biocompatibility, good mechanical properties, and excellent antibacterial capabilities [131]. Blended nanofiber membranes for new types of antibacterial wound dressings were made based on polycaprolactone/chitosan/*Aloe vera* (PCL/CS/AV) nanofiber (NFM) by electrospinning. The characterizations and tests carried out showed that the addition of *AV* increased the hydrophilicity and the pore size of the membranes and led to the improvement of the antibacterial performance against *Streptococcus aureus* and *E. coli* and the biocompatibility in 5 days. The membranes produced were proposed as suitable for short-term dressing or acute wounds (1–4 days) [132]. Nanofiber membranes were developed based on natural, biocompatible, and biodegradable composites from *AV* extract, pullulan, chitosan, and citric acid, through Forcespinning^®^ technology. The morpho-structural characterization and thermogravimetric analysis of the membranes indicated their good properties, as well as good water absorption capacities and synergistic antibacterial activity against *Escherichia coli,* which promoted cell attachment and growth. Due to their porous structure and large surface area, the membranes can be recommended as potential dressing applications due to their ability to absorb excessive blood and exudates, their thermal stability, and the protection they offer against infection [133]. Novel sodium alginate/poly(vinyl alcohol) (SA/PVA) hydrogel dressing films enriched with *AV* were produced by a simple method. The influence of different amounts (5, 10, 15, 20, and 25%, *v*/*v*) of *AV* solution on the chemical structure and properties of sodium alginate/poly(vinyl alcohol) hydrogel films was studied. The structural, morphological, mechanical, and thermal characterization confirmed that rigid and thermally stable three-dimensional structures were obtained. The results regarding the release profile of the polysaccharides from the hydrogel matrix showed that the active substance was released in a prolonged, gradual manner, even for a week. It was shown that the presence of *AV* within the cross-linked polymer network improved the active substance delivery properties of the hydrogel films. At the same time, the cytotoxicity of the materials was studied, and the results indicated good adhesion properties and a lack of toxicity. In vitro experiments on normal human dermal fibroblasts showed very good cell attachment to *AV* hydrogel discs, which promoted cell spreading and proliferation. As such, SA/PVA/*AV* sustained-release *AV* films have been proposed for applications such as interactive wound dressings [134]. Recent studies have concluded that *AV* gel is an effective antibacterial agent to prevent wound infection caused by various bacteria: *P. Aeruginosa* [135], *Campylobacter rectus*, *Provetella intermedia* [136], and *Escherichia coli* (*E. coli*) [137].

### 4.3. Skin Regeneration

**Figure 7:**
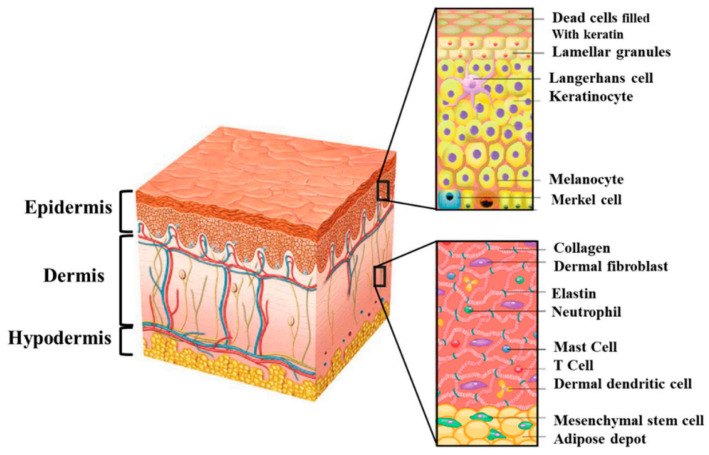
Structure of human skin: the epidermis (which contains keratinocytes, melanocytes, and Langerhans cells) and dermis (which includes fibroblasts, neutrophils, mast cells, and dermal dendritic cells), as well as subcutaneous hypodermis (which contains mesenchymal stem cells) [145].

The skin is part of the body’s integumentary system and consists of the epidermis and dermis, with a subcutaneous fatty layer, the hypodermis [138,139]. It protects us against external factors and prevents bacteria and germs from entering the body and blood and causing infections [140,141]. At the same time, the skin is vulnerable and can be affected by acute or chronic wounds [142]. Wound healing is a complex physiological process, which is achieved through four explicit phases: hemostasis, inflammation, proliferation, and remodeling and involves the epidermis-containing keratinocyte, melanocyte, and Langerhans cells, dermis, including fibroblast, neutrophil, mast cell, and dermal dendritic cells, and the hypodermis, which contains mesenchymal stem cells (Figure 7) [22,143,144,145].

**Figure 8:**
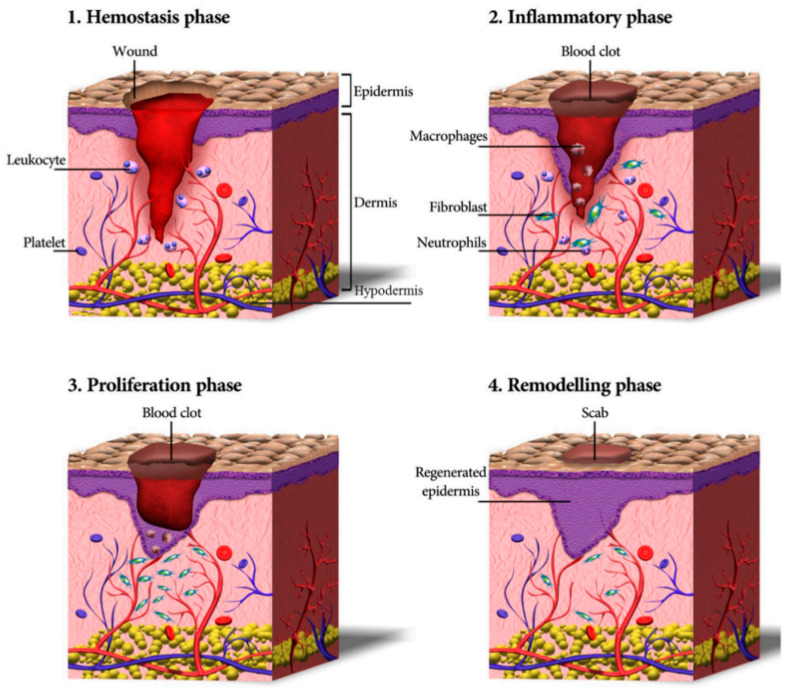
The phases of the wound-healing process [145].

The wound-healing process consists of four highly integrated and overlapping phases: (i) hemostasis, (ii) inflammation, (iii) proliferation, and (iv) tissue remodeling or resolution [146]. Figure 8 shows the main stages of the normal wound-healing process [145]. Each stage is characterized by key molecular and cellular events and is coordinated by a series of secreted factors that are recognized and released by wound response cells. Hemostasis is the first stage. It involves coagulation, which changes the blood from a liquid to a gel. The inflammation phase begins at the time of injury and lasts up to four days. As inflammatory cells undergo apoptosis, wound healing progresses to the proliferative phase. This phase begins approximately three days after the injury and overlaps with the inflammatory phase, while the tissue remodeling phase, characterized by the formation of granulation tissue, angiogenesis (formation of blood vessels), wound contraction, and the process of epithelialization, can continue for six months to one year after the injury, which leads to the formation of scar tissue. Many variables can disrupt one or more phases of this process, thereby producing inadequate or incorrect healing of skin wounds. The main elements that affect wound healing are oxygenation, infection, age, stress, diabetes, obesity, drugs, alcoholism, smoking, repeated trauma, diet, and poor blood circulation [147,148,149]. Infection is the most common complication for injured skin; therefore, prevention or mitigation of infection is of utmost importance. 

**Figure 9:**
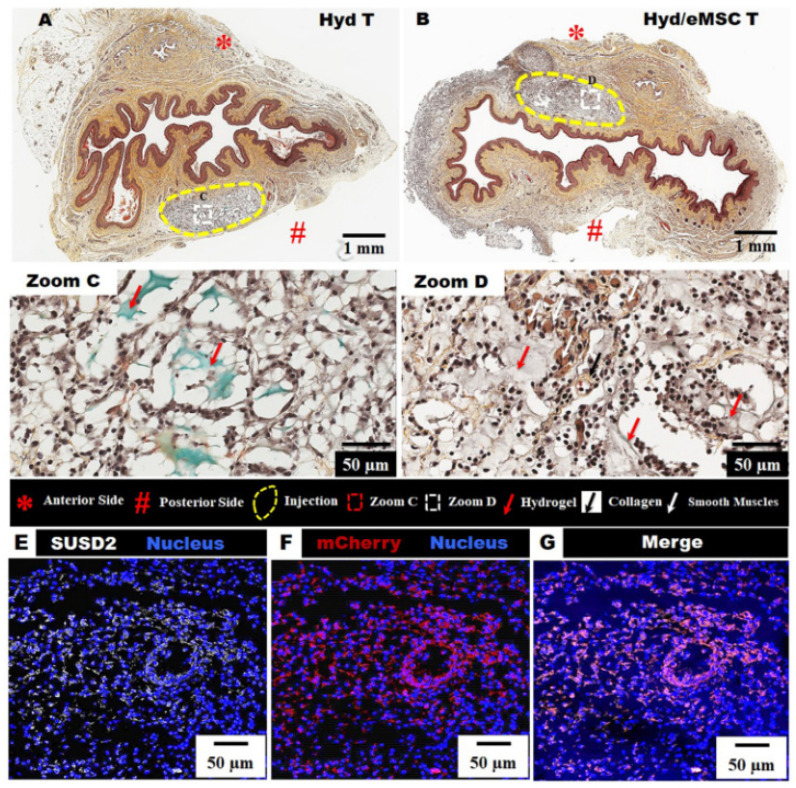
The retention of injected treatments with hydrogel and SUSD2 + mCherry + eMSC (**A**) Hyd T; (**B**) Hyd/eMSC T (yellow dotted lines); (**C**,**D**) red arrows—zoom area of hydrogel and black arrows—zoom area of collagen; (**E**) SUSD2, (**F**) mCherry, and (**G**) merge image of SUSD2 + mCherry in rat vaginal sections after 1 week. Reprinted with permission from ref. [151] Copyright 2023, Elsevier.

Using an ecological preparation method, a natural, degradable, and environmentally friendly hydrogel dressing was developed using *AV* as an active ingredient. The hydrogel dressing was prepared using only natural ingredients, composed of sodium hyaluronate (SH), dopamine (DA), chitosan (CS), and *AV*, and using a natural deep eutectic green solvent (DES) as the green solvent. The newly synthesized hydrogel showed good cytocompatibility tested on NIH-3T3 fibroblast cells and antibacterial properties against both Gram-positive (*S. aureus*) and Gram-negative (*E. coli*). Additionally, in a study on mice, the hydrogel promoted the regeneration of skin tissue and healed the skin wound after surgery within 12 days. The authors concluded that the newly prepared hydrogel, which is natural, degradable, and ecological and uses *AV* as an active ingredient, shows great potential in wound healing applications [150]. A study on the emergency treatment of vaginal tissue by local application of *AV* and alginate hydrogel for the release of mesenchymal stem cells derived from the maternal endometrium with the aim of promoting maternal injury relief and early healing was carried out in a simulated injury model at birth. It was observed that in the absence of therapy, fibrotic healing can occur in many cases. Local injection of hydrogel-containing mesenchymal cells significantly improves smooth muscle and elastin content, as well as decreases tissue stiffness after 6 weeks. The findings of the study highlighted that immediate treatment of severe vaginal birth trauma with therapeutic mesenchymal stem cells delivered in *AV* and alginate hydrogel might become a potential new treatment strategy for faster healing of birth injuries and prevention of pelvic organ prolapse (Figure 9) [151].

**Figure 10:**
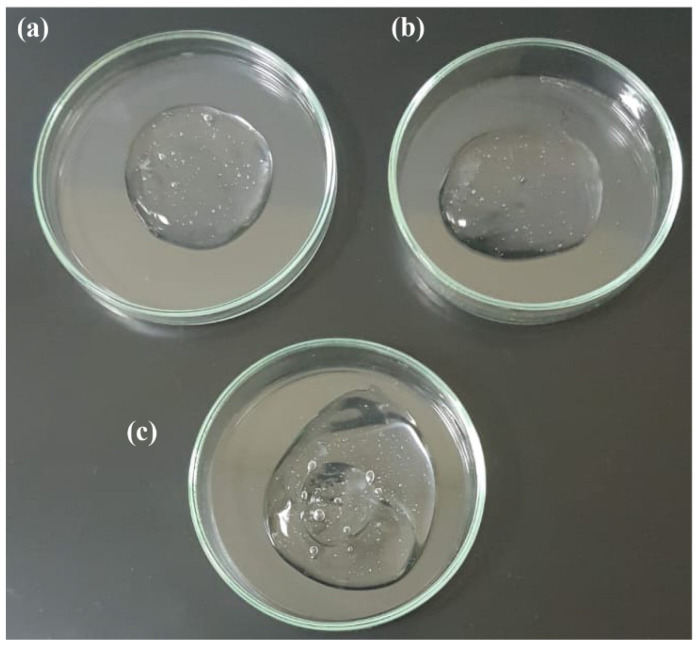
Images of different types of prepared hydrogel systems. (**a**) Hydrogel based only on Carbopol 934. (**b**) Placebo bioactive hydrogel (except piperine). (**c**) The bioactive hydrogel contains Carbopol 934, *Aloe vera*, and piperine [152].

**Figure 11:**
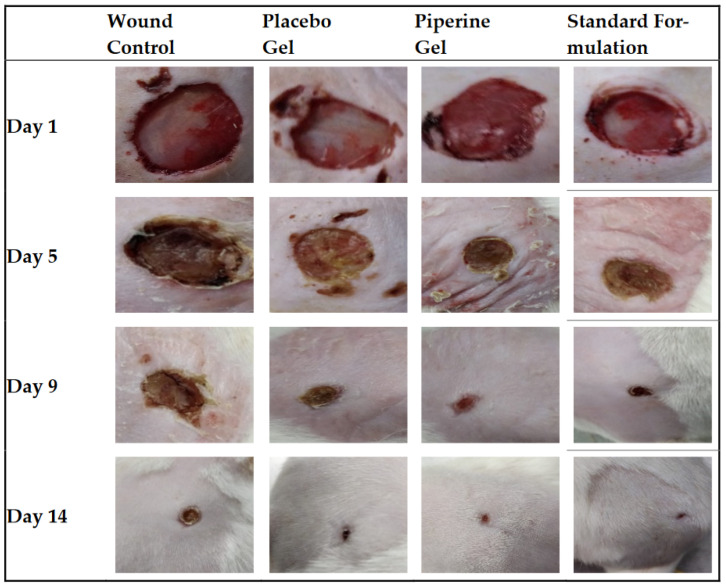
Phases of the wound-healing process in the untreated (control), placebo gel, piperine gel, and marketed standard formulation groups of rats. Photo for day 1, day 5, day 9, and day 14 of treatment [152].

Another study explored the potential for acute and chronic wound healing using piperine as a new bioactive compound. New systems of bioactive hydrogels based on carbopol 934 containing piperine mixed with *AV* gels of different gel strengths were prepared and characterized (Figure 10). The developed formulation system was investigated in an excisional wound healing model in the rat model. The results of the in vivo study and histopathological examination showed that the piperine-containing bioactive hydrogel system compared with the piperine-free bioactive hydrogel system, leads to early and intrinsic wound healing (Figure 11). Thus, the findings of the study emphasized that the new piperine-containing bioactive hydrogel is a promising therapeutic approach for the application of wound healing [152].

Studying the influence of a commercial hydrogel formulation based on *AV* with 1,2-propanediol (propanediol) and triethanolamine (TEA) on skin wound healing was investigated in female Wistar rats. Additionally, the study aimed to show that the presence of specific additives, propanediol and triethanolamine, does not exert any negative effect on wound healing.

**Figure 12:**
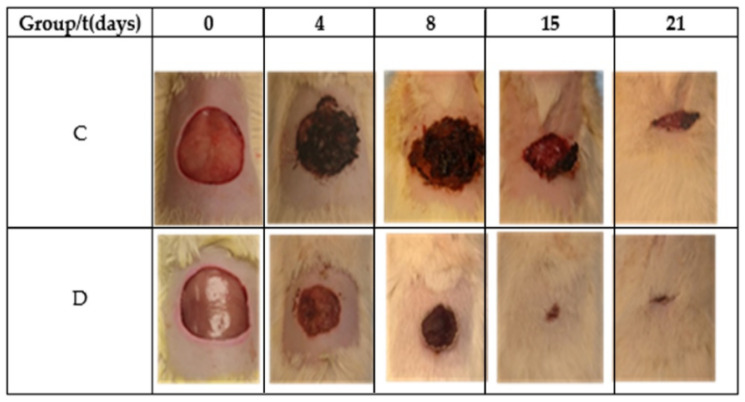
Images during the wound-healing process in female Wistar rats [153].

The results showed that the prepared hydrogel had a positive effect on inflammation, angiogenesis, and wound contraction and reduced the total healing time by 29%, with the total closure of the wound being achieved in 15 days (Figure 12). The paper highlighted the influence of the bioactive components of *AV*, related to rhamnogalacturonan and pectin-like acemannan, which improved the healing process of skin wounds [153].

**Figure 13:**
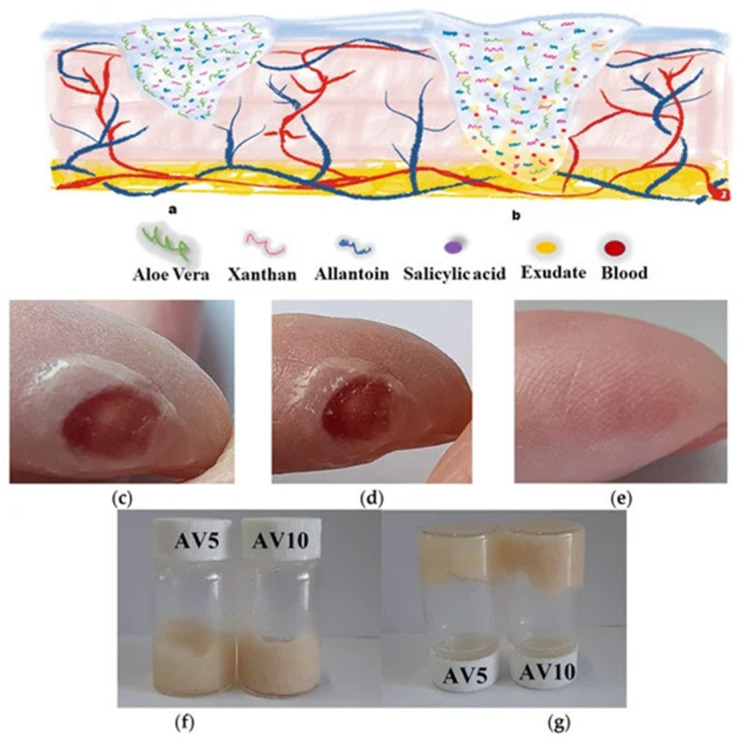
*Aloe vera*-based hydrogel for wound healing dressing: (**a**) dry hydrogel (**b**) wet hydrogel. The healing process (**c**) initial time; (**d**) after 5 min; (**e**) after 20 days; (**f**,**g**) inverted vial method [154].

A novelty in the area of efficient ecological materials is the new system of biocompatible hydrogels based on *AV* that was prepared by a completely green synthesis method for wound healing applications (Figure 13). 

**Figure 14:**
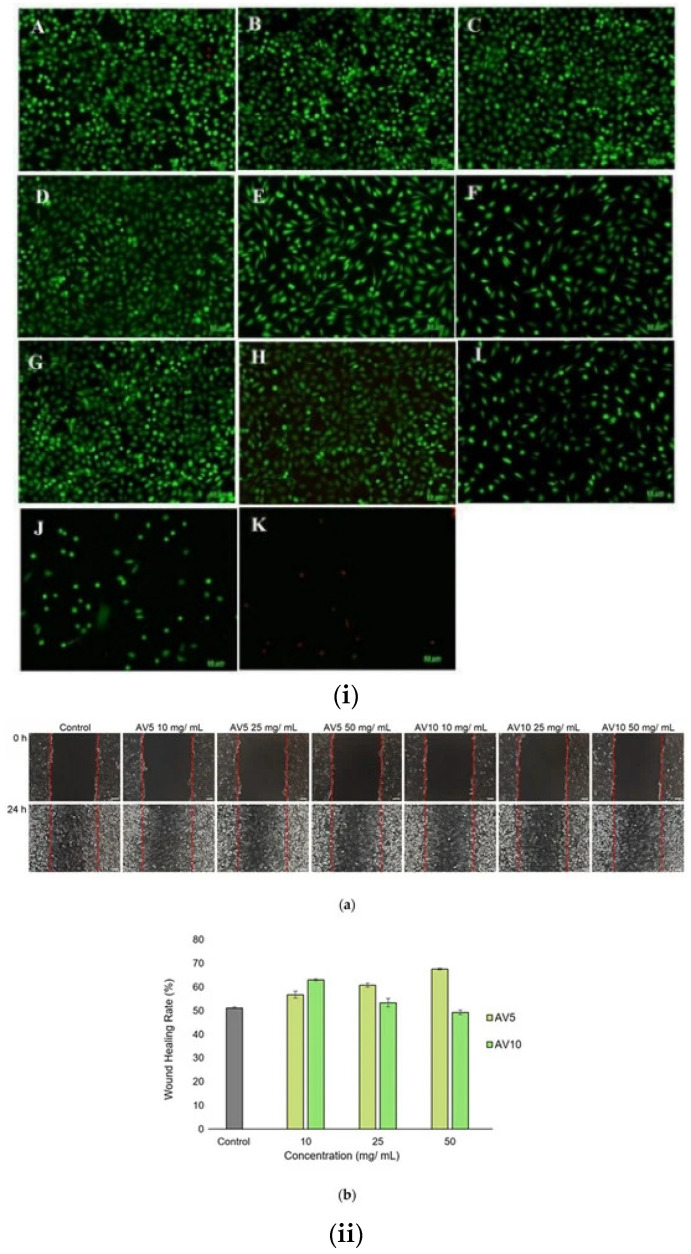
(**i**) Live/dead fluorescent images of L929, control, (**A**)—untreated and treated with AV5 (**B**–**F**) and AV10 (**G**–**K**) hydrogels at different concentrations for 48 h. (**B**,**G**)—10 mg/mL; (**C**,**H**)—25 mg/mL; (**D**,**I**)—50 mg/mL; (**E**,**J**)—75 mg/mL; (**F**,**K**)—100 mg/mL. (**ii**) Light microscope images (**a**) after in vitro generation of a wound for 24 h. (**b**) ImageJ analysis of wound closure percentage [154].

Hydrogels with different concentrations of *AV* (5 and 10%, respectively) also contain other natural components such as salicylic acid, allantoin, and xanthan gum. The hydrogels’ rheological properties, morphology, cell viability, biocompatibility, and cytotoxicity, were studied. The preliminary examinations showed that the hydrogels are very well supported on a wound, without stinging even more; they quickly penetrated the tissue and ensured good hydration of the area. Testing the antibacterial activity of the hydrogels was evaluated both on Gram-positive strains, *Staphylococcus aureus*, and on Gram-negative strains, *Pseudomonas aeruginosa*. The results showed that they have good antibacterial properties (Figure 14i). Moreover, the in vitro scratch test demonstrated the suitable ability of these “green” hydrogels to accelerate cell proliferation and migration and induce closure of a wounded area, making them suitable for wound healing applications (Figure 14ii) [154].

### 4.4. Healing Burns

A clinical study was conducted on 30 patients with similar types of second-degree burns in two places on different parts of the body. This research was conducted to evaluate the effectiveness of *AV* cream for partial thickness burns and to compare its results with those of silver sulfadiazine. Each patient had one burn treated randomly with topical silver sulfadiazine ointment and one treated with *Aloe* cream. The mean time to re-epithelialization and healing of partial-thickness burns was significantly shorter for the *Aloe* group at 15.9 ± 2 days versus 18.73 ± 2.65 days for the SSD group (*p* < 0.0001). Both sites were negative for microbial contamination on days 3, 7, and 13. Study results showed that *AV* cream promoted better wound healing with smaller lesions and had shorter healing times than silver sulfadiazine [155]. A similar international study was accomplished on 50 patients with second-degree burns and evaluated the effectiveness of *AV* gel compared with 1% silver sulfadiazine cream as a special dressing for the treatment of superficial and partial thickness burn wounds. The study used 98% unrefined gel from the inner leaf of the plant. Thermal burn patients bandaged with *AV* gel showed improvements compared to those bandaged with silver sulfadiazine cream in terms of early wound epithelialization, early pain relief, and cost-effectiveness of treatment management [156]. Another double-blind, randomized clinical trial in 11 patients treated once daily for 14 days compared the efficacy of herbal *AV* cream with 1% silver sulfadiazine in reducing the pain of second-degree burns. The herbal cream was prepared from *AV* gel and essential oils of *Lavandula stoechas* and *Pelargonium roseum*. In total, 56 patients were treated with herbal cream, and another 55 were treated with silver sulfadiazine 1%. Study results demonstrated that pain intensity at 14 days was significantly reduced in both groups compared to baseline (*p* < 0.001). However, a greater reduction in pain from baseline to the 7- and 14-day mark was observed in the herbal cream group (*p* = 0.014 and *p* = 0.05). One case of infection was reported in the herbal cream group; however, it cleared up with continued treatment. The findings of this clinical trial showed that the herbal cream was superior to silver sulfadiazine in relieving pain for superficial second-degree burns [157]. In an additional clinical case study, the therapeutic impact of *AV* gel on chronic skin burns in a 17-year-old patient with a rejected skin graft is presented. This is a before–after comparative study design in a case of fire burn in which initiation of *AV* gel treatment is accompanied by the promotion of wound repair. Before being treated with gel, the patient who had suffered burns on 30–40% of her body surface for 40 days had a healthy skin graft operation on her previous chest, which was rejected after 5 days. Following chronic unhealed skin lesions, the patient was treated with *AV* gel for 21 days continuously. The skin healing process began with the formation of granulation tissue and epithelization of the wounds. During the treatment, no sign of skin infection and no topical side effects of *AV* gel, such as allergic reactions and itching, were observed. This study on the impact of *AV* gel in the healing of burns can be considered a cheap and quick effect of substitution therapy instead of surgery [158].

### 4.5. Protection against Chemoradiation Secondary Effects in Cancer Treatment

A multicenter, randomized, double-blind, controlled trial was performed on 120 patients with head and neck cancer treated with concurrent chemoradiation. Patients received either *AV* gel or placebo gel and were assessed for adverse levels of skin toxicity with the Radiation-Induced Skin Reaction Rating Scale (RISRAS). At the 5th and 6th week of treatment, grades moderate to severe erythematous skin at values of 13.6% and 24.1% versus 27.8 and 42.6% were observed for members of the *AV* gel group and the placebo group, respectively (*p* = 0.05 for the 5th week and *p* = 0.038 for the 6th week). At week 7, in the placebo group, moderate to severe cases of wet scaling were observed in eight patients (19.0%) (*p* = 0.001), as well as a burning sensation with RISRAS scores of 3–4, representing only 11.9% of patients (*p* = 0.016). The study authors concluded that there was no prophylactic efficacy for radiation-induced dermatitis in the *AV* gel group compared with the placebo group but that topical applications of *AV* gel along with a routine skin care program from starting radiation would reduce the severity of any burning sensations, along with the incidence of erythematous, moist scaling of the skin in head and neck cancer patients receiving concurrent chemoradiation [159]. 

### 4.6. Summary of Clinical Effects of AV on Prevention and Healing of Skin Wounds

An earlier systematic study [121] concluded that *AV* helps to retain skin moisture and integrity and prevents skin ulcers due to its content of mucopolysaccharides, amino acids, zinc, and water. Furthermore, *AV* was found to be ‘much more effective and less costly compared to the currently available alternative treatments’ in terms of quality and speed of wound healing. Considering the tendency to promote traditional medicine as well as the rare side effects of AV, the use of this medicinal plant for the healing of skin wounds is recommended. 

AV gel has been demonstrated to be active in wound healing through several reported mechanisms [31], including increased epithelial cell viability, proliferation, and migration, moisture retention [160], increased quantity and cross-linking of collagen [161], and hindering inflammation through the decrease of proinflammatory cytokines [162,163,164,165,166]. The various active components of *AV* include acemannan, aloesin, aloe-emodin, aloin, emodin, and glucomannan [68]. Acemannan is known to stimulate epidermal keratinocytes and the production of fibrotic cytokines [167,168]. Glucomannan, a water-soluble mucopolysaccharide, stimulates fibroblast growth factor production and the activity and proliferation of these cells, leading to the increased amount of collagen on the wound site with enhanced transversal connections [21,64,169]. Emodin emodinolin, anthraquinone derivatives found in *AV*, act as competitive inhibitors of thromboxane synthetase and have significant anti-inflammatory properties [21]. The anti-inflammatory properties of *AV* are related to the inhibition of proinflammatory cytokines [162,164,165], hindering ROS production [162,164], and blocking the signalling of JAK1-STAT1/3 [68]. The anti-inflammatory effects and increased collagen production and cross-linking promote the rearrangement of epithelial tissues [12], reducing the wounded area and accelerating the healing process [170]. Various studies have confirmed that topical *AV* creams heal first- and second-degree burns in less than half the time than standard treatment with silver sulfadiazine [21,171,172,173]. *AV* has an anti-erythema activity similar to that of the positive control group (i.e., hydrocortisone gel) after 6 days of treatment [174]. *AV* gel has also demonstrated potent angiogenic activity, an essential process in wound healing, attributed to angiogenic compounds such as beta-sitosterol [175,176]. Table 2 summarizes various beneficial effects of *AV* compounds for wound healing reported in clinical studies.

**Figure 15:**
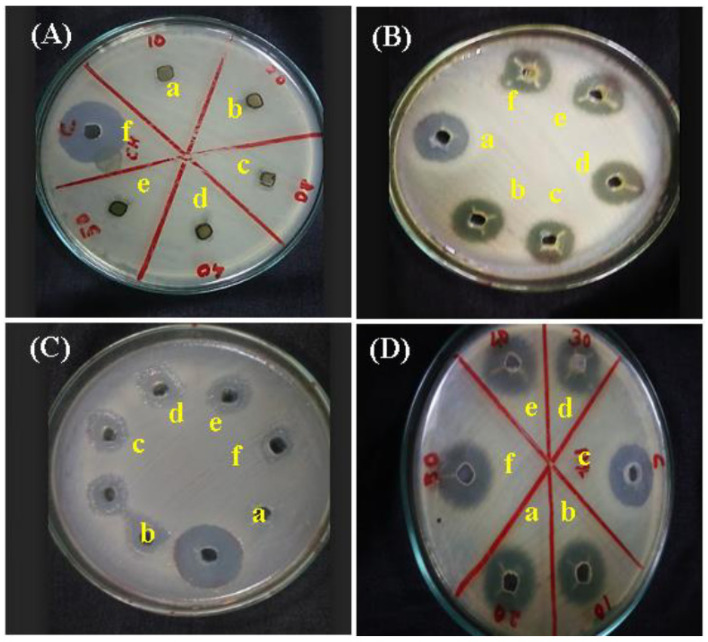
Agar assay (**a**) hydrogel; (**b**) hydrogel + GO; (**c**) hydrogel + rGO; (**d**) *Aloe vera* gel; (**e**) *Aloe vera* gel + GO; (**f**) *Aloe vera* gel + rGO against (**A**) *Pseudomonas aeruginosa*, (**B**) *Bacillus subtilis*, (**C**) *Staphylococcus aureus*, and (**D**) *E. coli*. [178].

Recent studies on *AV* gels with added therapeutic agents have reported the positive interaction between graphene oxide/reduced graphene oxide (GO/rGO) and *AV* hydrogels to be a strongly promising strategy for the advancement of therapeutic approaches for wound healing (Figure 15) [178].

**Figure 16:**
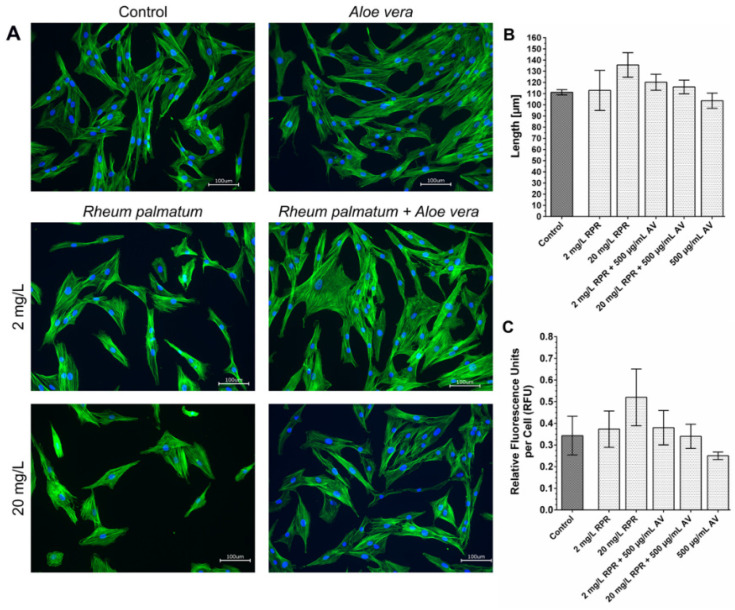
Actin staining of fibroblasts with phalloidin (**A**); the average length of the fibroblasts (**B**); fluorescent actin staining (**C**) [182].

Jales et al. further confirmed the great potential of *AV* mucilaginous hydrogel with a high keratolytic effect that can be used in psoriasis treatment [127]. Puliero et al. investigated the use of *AV* extracts for ocular therapeutic or preventive purposes. They demonstrated that the best lenses allowing for the high and stable release of *AV* extract to the corneal surface are those composed of ionic hydrogels [194]. Capsaicin, a powerful anti-inflammatory and analgesic agent, poorly water-soluble, was successfully incorporated into *AV* gel for topical drug delivery and to reduce skin irritation caused by capsaicin [119]. The *AV* gels softness, biocompatibility, and fast spreading or penetrating capacity are particularly useful features to encapsulate and deliver various nanoparticles with antimicrobial properties (e.g., ZnO or TiO_2_) [195], drugs, cell culture, both for wound healing, and bio-sensing applications [196]. The combination of *AV* and *Rheum palmatum* root can promote the migration of human primary fibroblasts (Figure 16) [182].

None of the dressings available on the market today are fully capable of reproducing all the characteristics of native skin. An asymmetric bilayer membrane with a top dense polycaprolactone layer that provides mechanical support and a bottom porous layer of chitosan and *AV*, aiming to improve the healing process, was designed to mimic both layers of the skin [197]. The results obtained revealed the potential of these asymmetric membranes to be applied as wound dressings in the future.

## 5. Side Effects

No serious adverse reactions were demonstrated following the topical application of *AV* inner gel products. *AV* used in dietary supplements appears to be safe [198]. The inner gel was evaluated by the Cosmetic Ingredient Review Expert Panel as noncytotoxic [199]. However, due to the cytotoxicity, mutagenicity, and carcinogenicity of anthraquinones, it is crucial to monitor the content of these phenolic compounds in *AV* whole leaf extract and latex [200,201]. Topical and oral use of *AV* whole leaf extract in humans can cause adverse clinical effects: skin irritation, hives, cramping, and diarrhea to those who are allergic to plants in the lily family, for example, onion and tulips [202,203,204].

## 6. Conclusions and Future Prospective Studies

It is important to apply modern delivery techniques to develop affordable products based on efficacious traditional natural medicines for wound healing and to improve their therapeutic effect.

Further research is needed to ensure that these formulations reach the pharmaceutical market. Chemotherapy treatments for cancer are associated with the presence of ulcers in the oral mucosa that causes pain, bleeding, and difficulty swallowing or speaking. There is no effective standard treatment, and few studies have been published on the therapeutic effects of natural products such as *AV* to improve the local retention period.

Future treatments may arise from medicinal plants, which have fewer side effects and improved bioavailability for the wound-healing process. In addition, in the future, a great challenge is represented by the development of an intelligent treatment that presents anti-inflammatory, antimicrobial, and antioxidant cumulative properties for the treatment of all types of wounds. Furthermore, the commercialization and use in preclinical research and clinical practice of natural products used in wound healing must be increased significantly to discover the potential of these products, considered natural bioactive molecules, in the treatment and regeneration of skin tissue. Future research should be considered to find new natural bioactive compounds related to their usage in the wound-healing process and their ability to act as substitutes for existing antibiotics.

By incorporating therapeutic agents into *AV*-based hydrogels, it is possible to develop multifunctional biomaterials that provide sustained release of agents, promote wound healing, reduce inflammation, and prevent or treat microbial infections. However, it is important to note that the specific formulation and efficacy of such hydrogels may vary depending on the therapeutic agents chosen, their concentration, crosslinking method, and other factors. Extensive research and testing (rheological analysis, drug release profiles, permeability, and stability studies) are required to optimize the formulation and ensure its safety and effectiveness for clinical use and to promote human well-being worldwide.

## Figures and Tables

**Table 1:** Compounds found in *Aloe vera* [64].

Type	Compounds
Anthraquinones/anthrones	Aloe-emodin, aloetic-acid, anthranol, aloin A and B (collectively known as barbaloin) isobarbaloin, emodin, ester of cinnamic acid
Carbohydrates	Pure mannan, acetylated mannan, acetylated glucomannan, glucogalactomannan, galactan, pectic substance, arabinogalactan, galactoglucoarabinomannan, galactogalacturan, xylan, cellulose, acemannan
Enzymes	Alkaline phosphatase, amylase, carboxypeptidase, carboxylase, catalase, cyclooxidase, phosphoenolpyruvate, cyclooxygenase, superoxide dismutase, lipase, oxidase
Inorganic compounds	Calcium, chlorine, phosphorous, chromium, copper, magnesium, iron, manganese, potassium, sodium, zinc
Non-essential and essential amino acids	Alanine, arginine, aspartic acid, glutamic acid, glycine, histidine, hydroxyproline, isoleucine, leucine, lysine, methionine, proline, threonine, tyrosine, valine, phenylalanine
Proteins	Lectins, lectin-like substance
Saccharides	Mannose, glucose, L-rhamnose, aldopentose,
Vitamins	B1, B2, B6, C, β-carotene, choline, folic acid, α-tocopherol
Miscellaneous	Arachidonic acid, γ-linolenic acid, potassium sorbate, steroids (campesterol, cholesterol, β-sitosterol), triglycerides, triterpenoid, gibberellin, lignins, salicylic acid, uric acid

**Table 2:** Beneficial effects for wound healing of *AV* gels.

Enhanced Reported	References
Cell viability	Sholehvar et al. [115], Liu et al. [177]
Epitelial cell proliferation	Moriyama et al. [167], Hashemi et al. [170], Shanmugan et al. [178], Teplicki et al. [179]
Epitelial cell midration	Teplicki et al. [179], Negahdari et al. [180], Wahedi et al. [181], Muller et al. [182]
Moisture retention	Dal’Belo et al. [160], Hamman et al. [183]
Keratinocyte proliferation	Moriyama et al. [167]
Collagen quantity	Hekmatpou et al. [21], Rahman et al. [64], Nabipour et al. [121], Abdel Hamid et al. [169], Hashemi et al. [170], Shanmugan et al. [178]
Collagen cross-linking	Hekmatpou et al. [21], Rahman et al. [64], Abdel Hamid et al. [169], Shanmugan et al. [178]
GSH activity	Liu et al. [177]
SOD activity	Liu et al. [177]
Antioxidant enzyme activity	Anilakumar et al. [184], Hassanpour et al. [185]
Accelerated wound healing	Moriyama et al. [167], Maenthaisong et al. [171], Somboonwong et al. [173], Shanmugan et al. [178], Negahdari et al. [180], Wahedi et al. [178,179,180,181], Hormozi et al. [186], Ali et al. [187]
Growth factors production	Hashemi et al. [170], Wahedi et al. [181]
Wound closure	Curto et al. [188]
Lysosomal stabilization	Paul et al. [165], DeOliveira et al. [189]
Stimulate fibrotic cytokines	Wahedi et al. [181], Zeng et al. [190]
Angiogenesis	Moon et al. [175], Choi et al. [176]
Block the signaling of JAK1-STAT1/3	Sánchez et al. [68]
Thromboxane reduction	Zeng et al. [21], Hekmatpou et al. [189]
Hindering IL-6	Ma et al. [162], Jiang et al. [164]
Hindering IL-8	Leng et al. [163], Na et al. [191]
Hindering IL-12	Ahluwalia et al. [163], Leng et al. [166]
TNF alpha levels reduced	Leng et al. [163], Jiang et al. [164], Paul et al. [165], Ahluwalia et al. [166]
Erythema reduction	Fox et al. [174], Reuter et al. [192]
Pain reduction	Hekmatpou et al. [21], Rompicherla et al. [119]
T cell proliferation suppressed	Li et al. [193]
Lipid peroxidation reduced	Liu et al. [177]
Proinflammatory cytokines reduced	Ma et al. [162], Leng et al. [163], Jiang et al. [164], Paul et al. [165], Ahluwalia et al. [166]
Type IV collagen degradation	Curto et al. [188]
ROS production hindered	Ma et al. [162], Jiang et al. [164]
Inflammation reduction	Hekmatpou et al. [21], Paul et al. [165]

## Data Availability

Not applicable.
